# Increased medial prefrontal cortical thickness and resilience to traumatic experiences in North Korean refugees

**DOI:** 10.1038/s41598-021-94452-6

**Published:** 2021-07-21

**Authors:** Hyunwoo Jeong, Yu Jin Lee, Nambeom Kim, Sehyun Jeon, Jin Yong Jun, So Young Yoo, So Hee Lee, Jooyoung Lee, Seog Ju Kim

**Affiliations:** 1 Geumsan-Gun Public Health Center, Geumsan, Republic of Korea; 2 Department of Psychiatry and Center for Sleep and Chronobiology, Seoul National University Hospital, Seoul, Republic of Korea; 3 Neuroscience Research Institute, Gachon University, Incheon, Republic of Korea; 4 Department of Psychiatry, Korea University College of Medicine, Korea University Anam Hospital, Seoul, Republic of Korea; 5 Department of Psychiatry, Seoul National Hospital, Seoul, Republic of Korea; 6 Department of Psychiatry, National Medical Center, Seoul, Republic of Korea; 7 Department of Psychiatry, Sungkyunkwan University School of Medicine, Samsung Medical Center, Seoul, Republic of Korea

**Keywords:** Psychology, Anxiety, Depression, Post-traumatic stress disorder, Brain

## Abstract

Little is known regarding structural brain changes in traumatized refugees and the association with psychopathology. In the present study, the cortical thickness in North Korean refugees and the association with psychological symptoms were explored. North Korean refugees with lifetime post-traumatic stress disorder (PTSD group, n = 27), trauma-exposed North Korean refugees without lifetime PTSD (trauma-exposed control (TEC) group, n = 23), and healthy South Korean controls without traumatic experiences (HC group, n = 51) completed questionnaires assessing depression, anxiety, somatization, and PTSD symptoms. The cortical thickness was measured by magnetic resonance imaging (MRI) using FreeSurfer. Age- and sex-adjusted cortical thickness of the right medial prefrontal cortex (mPFC) was greater in the TEC group than in the HC group. However, significant differences were not observed between the PTSD and HC groups. Increased right mPFC thickness was significantly correlated with less anxiety and somatization after controlling for age and sex in the TEC group, but not in the PTSD or HC groups. North Korean refugees who did not develop PTSD after trauma showed increased right mPFC thickness, which was associated with less severe psychiatric symptoms. These findings indicate that increased mPFC thickness might have helped to reduce PTSD and psychiatric symptoms after trauma, and likely reflects resilience achieved by potentially enhancing emotional regulation in the mPFC.

## Introduction

More than 1% of humanity is now forcibly displaced due to persecution, violence, or human rights violations, and the world’s refugee population continues to increase in size^1^. Refugees are often exposed to multiple traumatic events, and accordingly show a high prevalence of mental health problems such as post-traumatic stress disorder (PTSD), anxiety, depression, and somatization^2,3^. Identifying neurobiological correlates of psychopathology in traumatized refugees is important because this may facilitate effective prevention of, and interventions for, psychiatric illness in this population^4^.

The brain structures of traumatized refugees have been investigated in only two previous studies. Vietnamese ex-political detainees reportedly showed a trend toward lower cortical thickness in some prefrontal and temporal regions, compared with non-traumatized controls^5^. In another study, traumatized refugees had smaller lateral prefrontal, parietal, and posterior midline structure volumes compared with healthy controls, and the reduction was more prominent in refugees with PTSD than in those without PTSD^6^. Despite these studies, the characteristics of cortical thickness in refugees remain unclear; inconclusive results were reported in the former study, while the latter was conducted on gray matter volume, which correlates poorly with cortical thickness^7^.

Numerous structural imaging studies have been conducted in other traumatized populations; most of these compared cortical thickness or gray matter volume between traumatized individuals with and without PTSD. The results showed reduced cortical thickness or gray matter volume mostly in frontotemporal areas in subjects with PTSD^8–12^. However, separate studies in refugees may enhance the understanding of this population, because their experiences differ relative to those related to other types of trauma; moreover, outcomes vary among trauma types^13^. Most refugees are exposed to traumatic events throughout their lifetime due to political or religious oppression, war, and migration^14,15^. Refugees may experience imprisonment, torture, physical assault, and rape before fleeing; they are frequently robbed, witness torture or killing, lose close family members or friends, and endure harsh environmental conditions during the flight process^15^. Some experiences may not be consistent across traumatized individuals, such as challenges associated with migration to another country, adjustment to a new culture, and changes in social roles. In addition, the large breadth of trauma in refugees is supported by a study showing an average of 150 traumatic events per person^16^. These broad traumatic experiences may affect the cortical structures of refugees, because lifetime trauma burden has been suggested to have a cumulative effect on cortical thickness^17^. Furthermore, a non-traumatized healthy control group was not included in most of the previous studies, which is crucial in cross-sectional designs to determine whether brain structural differences among traumatized individuals are associated with vulnerability or resilience^18^. The cortical thickness in traumatized individuals was compared with healthy controls in only a few studies, and the results were inconsistent; increased cortical thickness in prefrontal or temporal regions was reported in some studies^19,20^, while widespread cortical thickness reduction was reported in another study^21^.

Due to the paucity of conclusive findings in previous studies, the cortical thickness in refugees with PTSD, refugees without PTSD, and healthy South Korean controls without exposure to trauma were compared in the present study. In addition, the association between cortical thickness and psychiatric symptoms, such as anxiety, depression, and somatization, were investigated. Based on previous studies, we hypothesized that there would be differences in cortical thickness among the three groups, and that cortical thickness would correlate with psychiatric symptoms in the North Korean refugees.

## Methods

### Participants

North Korean refugees residing in South Korea and native South Korean residents were recruited to the present study through advertisements. Individuals were excluded if they had a history of serious head injury, neurological disorder, serious untreated medical illness, neurodevelopmental disorder, and/or any metal or electronic device inside the body. Initially, 54 North Korean refugees and 55 South Korean residents were recruited to the study. All North Korean refugee subjects were exposed to traumatic events at least once in their lifetime, while no South Korean resident subjects had any lifetime exposure to trauma, nor any history of psychiatric disorders. Among the subjects, three North Korean refugees were excluded due to anatomical abnormalities in their brain, and one North Korean refugee and four South Korean residents were excluded due to poor image quality. Thus, the final study sample consisted of 50 North Korean refugees and 51 South Korean residents. Among the refugees, 27 were diagnosed with lifetime PTSD (based on the DSM-IV-TR criteria^22^) and were assigned to the PTSD group. The remaining 23 refugees did not meet the diagnostic criteria for PTSD and were assigned to the trauma-exposed control (TEC) group. The healthy control (HC) group consisted of 51 South Korean residents.

This study was approved by the Institutional Review Board of Seoul National University Hospital and all participants provided written informed consent before participating. All participants were paid for their participation. All procedures in this study were performed in accordance with the Declaration of Helsinki, which describes the ethical principles for medical research involving human subjects.

### Clinical evaluation

All participants were evaluated using the Korean version of the Structured Clinical Interview for DSM-IV Axis I Disorders (SCID)^23^ for diagnoses of lifetime PTSD among North Korean refugees and to exclude psychiatric disorders among South Korean residents. Lifetime psychiatric comorbidities were also assessed using the SCID. The Korean version of the SCID has been demonstrated to have good interrater reliability, with Kappa coefficients greater than 0.70 for most disorders^23^.

Korean versions of the Beck Anxiety Inventory (BAI)^24^, Beck Depression Inventory (BDI)^25^, and Symptom Checklist-90-Revised (somatization symptom items only; SCL-somatization)^26^ were also administered to each participant to assess the severities of anxiety, depression, and somatization, respectively. Refugees were administered the Impact of Event Scale-Revised (IES-R)^27^ to assess symptom severity in three PTSD domains: intrusion, avoidance, and hyperarousal. Total IES-R scores and all three subscale scores were analyzed. Korean versions of the BAI (Cronbach α = 0.91), BDI (Cronbach α = 0.88), SCL-somatization (Cronbach α = 0.94), and IES-R (Cronbach α = 0.93) reportedly have high internal consistency^24,25,27,28^. Korean versions of the BAI, BDI, and IES-R have been demonstrated to correlate well with other measures related to anxiety, depression, and PTSD, respectively^24,25,27^.

The refugees’ previous exposures to traumatic events were evaluated using the Trauma Exposure Check List for North Korean Refugees^29^, which assesses whether respondents were exposed to any of 13 types of traumatic events during residency in North Korea or to any of 16 types of traumatic events during their escape prior to their arrival in South Korea. The traumatic events listed in the questionnaire comprise either direct or vicarious exposure to torture, severe battery, life-threatening starvation/cold/accidents, rape, human trafficking, arrest/incarceration, and witnessing of public executions; all included events meet the DSM-IV criteria for PTSD trauma. The number of trauma types experienced was counted for each respondent as a surrogate measure for trauma burden. The refugees’ duration of habitation in South Korea and number of years of education were also assessed. Medication use among the refugees was evaluated by interviewing the participants. Because previous studies have mostly reported brain structure alterations associated with psychotropic drugs^30,31^, psychotropic and non-psychotropic medication use were assessed separately.

### Magnetic resonance imaging (MRI) data acquisition

Subjects underwent high-resolution structural imaging using a 3 T magnetic resonance imaging (MRI) system (Trio Tim; Siemens, Erlangen, Germany) with a 12-channel birdcage head coil. The 3D magnetization-prepared rapid gradient echo scan was obtained with the following imaging parameters: TR = 1,670 ms, TE = 1.89 ms, TI = 900 ms, flip angle = 9°, slice thickness = 1.0 mm, in-plane resolution = 1.0 × 1.0 mm^2^, FOV = 250 mm, and matrix size = 256 × 256.

### Data analyses

Group differences in categorical variables were tested using the chi-squared test or Fisher’s exact test, as appropriate. When differences among the three groups were statistically significant, post hoc pairwise comparisons were performed using the Z-test or Fisher’s exact test, as appropriate. For continuous variables, the three groups were compared using one-way analysis of variance (ANOVA) with the post hoc Tukey test, or Welch’s ANOVA with the post hoc Games-Howell test, as appropriate. Independent *t*-tests were used to compare total and subscale IES-R scores, the number of trauma types experienced, the duration of habitation in South Korea, and the number of years of education between the PTSD and TEC groups.

Cortical reconstructions of the T1-weighted images from all subjects were performed using FreeSurfer software (version 7.1.1; http://surfer.nmr.mgh.harvard.edu)^32^. Every coronal slice of each subject’s scan was visually examined for accuracy of the gray/white matter boundary. Manual edits were applied as necessary and scans were excluded from the study if edits were impossible due to poor image quality. Cortical thickness was measured by calculating the shortest distance between the pial surface and white matter at each vertex. Each subject’s reconstructed brain was then registered to a spherical space based on individual cortical folding patterns to match cortical geometry across subjects, and each subject’s cortical thickness measurements were assembled onto this new space. The cortical thickness maps were spatially smoothed with a Gaussian kernel (10 mm full-width at half-maximum) before further analyses.

Cortical thickness was compared among the three study groups using the general linear model and corrected for multiple comparisons via nonparametric permutation cluster analysis (cluster-forming threshold: *P* < 0.05; cluster-wise *P*-value: *P* < 0.05, two-tailed). This group analysis was controlled for age because the three study groups differed significantly in that parameter, which may have played a role in the group differences in cortical thickness given the association between aging and cortical thinning^33^. Each participant’s cortical thickness for the identified cluster was extracted from FreeSurfer, and one-way analysis of covariance (ANCOVA) was performed to test for group differences in the cortical thickness of the cluster while controlling for age and sex. Post hoc pairwise comparisons with the Bonferroni correction of age- and sex-adjusted cortical thickness were performed between groups in the cluster identified from the initial group analysis. Secondary exploratory partial correlation analyses, controlled for age and sex, were performed within each study group to identify associations between the cortical thickness in the identified cluster and the clinical variables (e.g., BAI, BDI, SCL-somatization, and IES-R). Correlations between the number of trauma types experienced and cluster thicknesses within the PTSD, TEC, and total refugee (PTSD + TEC) groups were also calculated. Cortical reconstruction, preprocessing, and permutation cluster analysis were performed using FreeSurfer, and the remaining statistical analyses were performed using R software (version 4.0.2)^34^. A two-tailed *P*-value < 0.05 was considered statistically significant.

## Results

### Demographic and clinical characteristics

The demographic and clinical characteristics of the participants are summarized in Table 1. Although there were no significant group differences in sex, the groups differed in age [F(2, 98) = 4.8, *P* = 0.01], BAI score [F(2, 35.9) = 24.3, *P* < 0.001], BDI score [F(2, 41.8) = 10.5, *P* < 0.001], and SCL-somatization score [F(2, 35.7) = 17.0, *P* < 0.001]. The mean age was significantly higher in the PTSD group (40.3 ± 11.5 years) than in the TEC group (30.9 ± 8.1 years; *P* = 0.007); the mean age in the HC group (36.6 ± 11.2 years) was not significantly different from the mean ages in the other two groups. BAI, BDI, and SCL-somatization scores were higher in the PTSD group (BAI = 28.3 ± 13.2, BDI = 23.0 ± 14.6, SCL-somatization = 31.2 ± 11.6) than in the TEC group (BAI = 10.9 ± 9.2, *P* < 0.001; BDI = 8.4 ± 8.3, *P* < 0.001; SCL-somatization = 20.2 ± 7.8, *P* = 0.002) and the HC group (BAI = 7.1 ± 7.6, *P* < 0.001; BDI = 8.5 ± 8.7, *P* < 0.001; SCL-somatization = 16.5 ± 4.1, *P* < 0.001); significant differences were not observed between the TEC and HC groups. Total IES-R scores, the three IES-R subscale scores, and the number of trauma types experienced were higher in the PTSD group than in the TEC group. The duration of habitation in South Korea and the number of years of education did not differ between the two groups.

**Table 1:** Demographic and clinical characteristics of the participants.

Variable	PTSD group (n = 27)	Trauma-exposed controls (n = 23)	Healthy controls (n = 51)	*P*-value
Age (years)	40.3 (11.5)	30.9 (8.1)	36.6 (11.2)	0.01
**Sex**				0.32
Male (n) (%)	4 (14.8%)	7 (30.4%)	15 (29.4%)	
Female (n) (%)	23 (85.2%)	16 (69.6%)	36 (70.6%)	
BAI	28.3 (13.2)	10.9 (9.2)	7.1 (7.6)	< 0.001
BDI	23.0 (14.6)	8.4 (8.3)	8.5 (8.7)	< 0.001
SCL-somatization	31.2 (11.6)	20.2 (7.8)	16.5 (4.1)	< 0.001
**IES-R**				
Total	35.4 (20.2)	10.6 (12.5)	-	< 0.001
Intrusion	13.7 (8.5)	4.6 (5.1)	-	< 0.001
Avoidance	13.3 (7.0)	3.4 (4.4)	-	< 0.001
Hyperarousal	10.4 (6.1)	2.9 (3.5)	-	< 0.001
Number of trauma types experienced	6.1 (3.2)	3.5 (2.3)	-	0.003
Duration of habitation in South Korea (months)	61.8 (28.7)	68.3 (37.8)	-	0.52
Number of years of education	11.5 (3.6)	12.9 (3.9)	-	0.21
Participants on psychotropic medications (n) (%)	6 (22.2%)	0 (0%)	-	0.03
Participants on non-psychotropic medications (n) (%)	6 (22.2%)	1 (4.3%)	-	0.11

Continuous variables are presented as mean (standard deviation).

*PTSD* post-traumatic stress disorder, *BAI* beck anxiety inventory, *BDI* beck depression inventory, *SCL-somatization* somatization subscale of Symptom Checklist-90-Revised, *IES-R* impact of event scale-revised.

The refugees reported using psychotropic drugs including antidepressants and sedatives. They also reported using non-psychotropic drugs for conditions including uterine tumors, chronic urticaria, cardiac disease, osteoarthritis, and headaches. Six participants in the PTSD group were on psychotropic medications, whereas no participant in the TEC group was on any psychotropic medication. Six participants from the PTSD group were on non-psychotropic medication, whereas one participant from the TEC group was on non-psychotropic medication. The PTSD and TEC groups differed in the prevalence of psychotropic medication use (*P* = 0.03) but not in the prevalence of non-psychotropic medication use (*P* = 0.11).

Lifetime psychiatric comorbidities of the participants are summarized in Supplementary Table S1. The comorbidities were classified into the following five categories: anxiety disorders, depressive disorders, somatic symptom and related disorders, adjustment disorders, and feeding and eating disorders. Anxiety disorders diagnosed in the participants included generalized anxiety disorder, panic disorder, and specific phobia. Depressive disorders included major depressive disorder and dysthymia. Somatic symptom and related disorders included somatic symptom disorder; feeding and eating disorders included bulimia nervosa. The three groups exhibited significant differences in the prevalence of anxiety disorders, depressive disorders, somatic symptom and related disorders, and adjustment disorders; however, there were no significant group differences in the prevalence of feeding and eating disorders. Post hoc pairwise comparisons revealed that the prevalence of depressive disorders was higher in the PTSD group than in the TEC (*P* = 0.01) or HC (*P* < 0.001) group, whereas there was no significant difference between the TEC and HC groups (*P* = 0.93). The prevalence of anxiety disorders and somatic symptom and related disorders was higher in the PTSD group than in the HC group (anxiety disorders: *P* = 0.003; somatic symptom and related disorders: *P* < 0.001), but there were no significant differences observed in comparisons of other pairs of groups (anxiety disorders: PTSD *vs.* TEC, *P* = 0.32; TEC *vs.* HC, *P* = 0.93; somatic symptom and related disorders: PTSD *vs.* TEC, *P* = 0.08; TEC *vs.* HC, *P* = 0.93). No significant difference in the prevalence of adjustment disorders was found in all pairwise comparisons (PTSD *vs.* TEC, *P* = 1.00; PTSD *vs.* HC, *P* = 0.11; TEC *vs.* HC, *P* = 0.93).

### Group differences in cortical thickness

**Figure 1:**
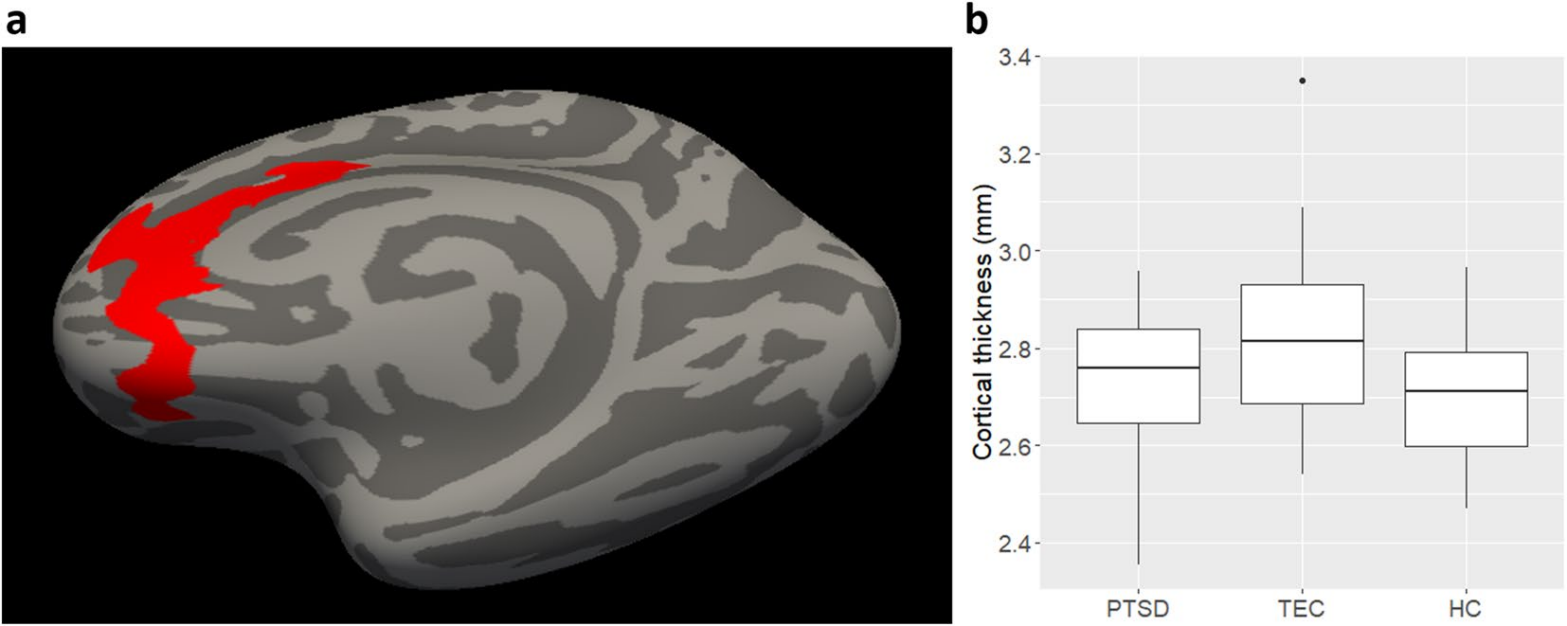
Cluster analysis showing significant differences in cortical thickness among the post-traumatic stress disorder, trauma-exposed controls, and healthy control groups. (**a**) Cluster projected onto the inflated surface of the right hemisphere. (**b**) Cortical thickness of the cluster for all groups. In the box plots, lines within the boxes denote median values, boxes extend from the 25th to the 75th percentile, whiskers denote adjacent values (i.e., the most extreme values within 1.5 times the interquartile range of the 25th and 75th percentiles), and dots denote outliers outside the range of adjacent values. *PTSD* post-traumatic stress disorder group, *TEC* trauma-exposed controls, *HC* healthy controls.

Permutation cluster analysis identified a single cluster in the right medial prefrontal cortex (mPFC), in which cortical thickness differed significantly among the three study groups (cluster size = 1,855.22 mm^2^, cluster-wise *P* = 0.008; Fig. 1). The right mPFC cluster spanned the anterior cingulate cortex (ACC) and the ventromedial prefrontal cortex (vmPFC). In the study groups, the means and 95% confidence intervals (CIs) for the unadjusted cortical thickness in the right mPFC cluster were as follows: PTSD, 2.74 mm (95% CI 2.68–2.80); TEC, 2.82 mm (95% CI 2.74–2.90); HC, 2.70 mm (95% CI 2.67–2.74). The age- and sex-adjusted estimated marginal means and 95% CIs for the right mPFC cortical thickness were as follows: PTSD, 2.76 mm (95% CI 2.71–2.81); TEC, 2.80 mm (95% CI 2.74–2.85); HC, 2.70 mm (95% CI 2.67–2.74). A significant difference in the cortical thickness of the right mPFC cluster was discovered among groups while controlling for age and sex [F(2, 96) = 3.70, *P* = 0.03]. Post hoc pairwise comparisons showed that the age- and sex-adjusted cortical thickness was higher in the TEC group than in the HC group (*P* = 0.04), however, the differences between the other group pairs were not statistically significant (PTSD *vs.* TEC: *P* = 1.00; PTSD *vs.* HC: *P* = 0.35).

### Associations between cortical thickness of the right mPFC cluster and clinical features

**Figure 2:**
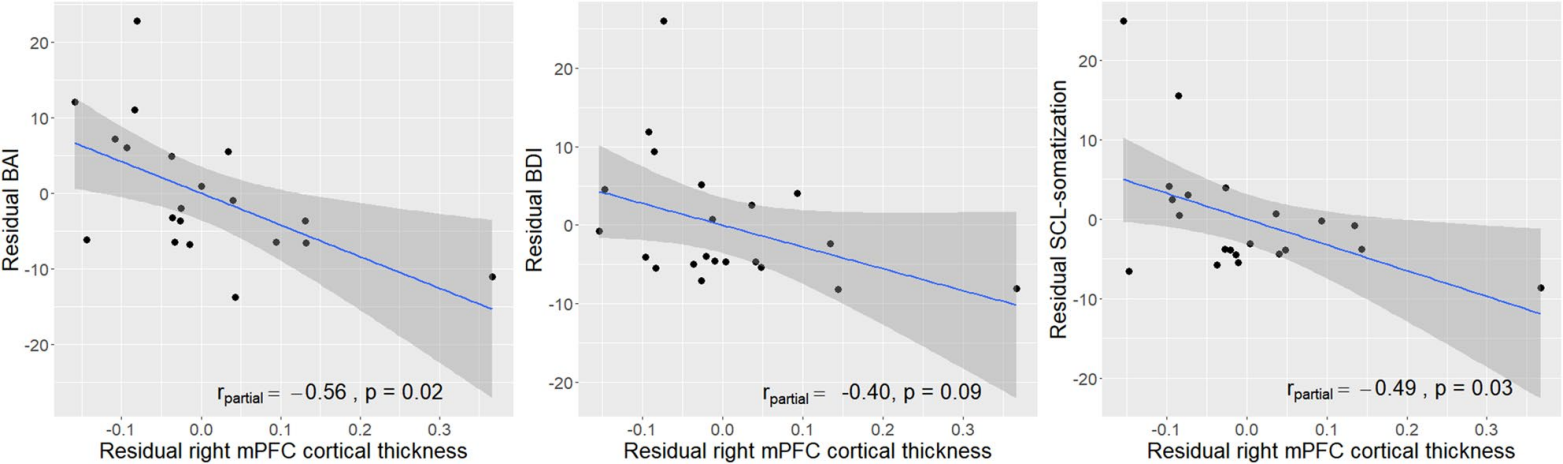
Negative correlations between psychiatric symptom scores and the cortical thickness of the right mPFC cluster in the trauma-exposed control group after adjusting for age and sex. *mPFC* medial prefrontal cortex, *BAI* beck anxiety inventory, *BDI* beck depression inventory, *SCL-somatization* somatization subscale of Symptom Checklist-90-Revised.

In the TEC group, the cortical thickness of the right mPFC cluster was negatively correlated with the BAI (r_partial_ =  − 0.56, *P* = 0.02), BDI (r_partial_ =  − 0.40, *P* = 0.09), and SCL-somatization (r_partial_ =  − 0.49, *P* = 0.03) scores after adjusting for age and sex (Fig. 2), although the statistical significance of the BDI correlation was slightly below the threshold level. Significant correlations of the right mPFC thickness with the total and subscale IES-R scores were not found in the TEC group. The right mPFC thickness was not significantly correlated with any of the clinical variables in the PTSD or HC groups.

There were no significant correlations between the right mPFC thickness and number of trauma types experienced in the total refugee group (PTSD + TEC) or each group (PTSD + TEC: r =  − 0.10, *P* = 0.50; PTSD: r =  − 0.14, *P* = 0.51; TEC: r = 0.17, *P* = 0.47).

## Discussion

In the present study, TECs showed greater right mPFC thickness than did refugees with PTSD. The greater mPFC thickness was associated with less severe anxiety, depression, and somatization in TECs, but not in refugees with PTSD or HCs.

As expected, the severities of anxiety, depression, somatization, and PTSD symptoms were higher in the PTSD group. The TECs and HCs showed no significant differences in these psychological symptoms. Similarly, the prevalence of anxiety disorders, depressive disorders, and somatic symptom and related disorders was higher in the PTSD group than in HCs, whereas TECs did not differ significantly in the prevalence relative to HCs. The prevalence of feeding disorders was comparable among groups, and no significant differences in the prevalence of adjustment disorders was seen for any group pair. These findings indicate that the TECs were resilient to trauma and capable of maintaining general mental health at a level similar to HCs.

Both the PTSD and TEC groups experienced multiple types of trauma, consistent with previous reports demonstrating multiple traumatic exposures in refugees^16^. The duration of habitation in South Korea did not differ between the PTSD and TEC groups, suggesting that the time since exposure to trauma would be comparable between the two groups. The measured duration could roughly reflect time since exposure to trauma, because refugees are usually safe after they resettle in South Korea. Both groups had a considerable level of education, with the length of education comparable between the two groups.

Consistent with our primary hypothesis, significant differences were found in cortical thickness in the right mPFC among groups. Only TECs had a thicker mPFC compared with HCs, and mPFC thickness was not significantly different between the refugees with PTSD and HCs. The lack of correlation between the number of trauma types experienced and the mPFC thickness suggests that the cortical thickness difference among groups was not confounded by the difference in number of trauma types experienced among groups. Data regarding the education levels of HCs were lacking, but it is unlikely that gaps in education level would have caused the cortical thickness difference among groups. If gaps in education level existed among the groups, HCs would likely have had a higher level of education compared with the PTSD or TEC group, considering that South Korea has one of the highest rates of tertiary education attainment worldwide^35^. This would be associated with reduced cortical thickness in the PTSD and TEC groups, given that a lower level of education is reportedly related to thinner cortical thickness^36^. However, the results of the current study revealed greater or equivalent cortical thicknesses in the two refugee groups compared with HCs, which cannot be explained by gaps in education level among the groups.

Because the identified right mPFC cluster spanned the ACC and vmPFC, the current findings suggest that increased ACC or vmPFC thicknesses in traumatized refugees may represent neurobiological resilience to trauma. Increased cortical thicknesses of the ACC and vmPFC have been suggested to be associated with resilience to traumatic experiences in diverse groups of trauma victims. Greater ACC thickness was reportedly associated with improvement in PTSD patients over time^37^. In a study of children with PTSD, a thicker vmPFC was associated with less severe PTSD^38^. In addition, sexual trauma victims without PTSD exhibited greater mPFC recruitment during negative emotion regulation tasks than did victims with PTSD and HCs^39^. Both the ACC and vmPFC modulate negative emotions via top-down control over the amygdala^18^. The increased mPFC thickness in TECs may have helped prevent the development of PTSD by enabling better regulation of negative emotions after traumatic experiences.

Thicker ACC and vmPFC are reportedly associated with lower anxiety in patients with anxiety disorder and healthy individuals^40–42^. Moreover, a better response to depression treatment was reportedly correlated with a greater increase in ACC thickness over time^43,44^. A thicker ACC was reportedly associated with a better ability to manage sadness among healthy family members of depressed patients^45^. Relationships of ACC thickness with both anxiety and depression were also reported among Cushing’s disease patients with long-term remission^46^. Consistent with all of these previous reports, a thicker mPFC was correlated with less anxiety and depression in our TECs, which supports the emotional regulatory role of the mPFC in these refugees.

Somatization is strongly associated with anxiety and depressive symptoms^47^. Because negative emotions, such as low mood or anxiety, may amplify an individual’s perception of visceral or somatic sensations, somatization may be mediated by cortical structures associated with emotional processing, including the ACC and vmPFC^48^. In agreement with this theory, decreased ACC/vmPFC gray matter and its relationship with symptom severity have been reported in patients with somatoform disorder^49,50^. In the present study, a negative correlation was found between right mPFC thickness and somatization in TECs, in agreement with previous studies.

Due to the cross-sectional design of the current study, definitive conclusions cannot be drawn regarding whether the increased right mPFC thickness in TECs developed after trauma due to neuroplastic compensation, or whether it existed before trauma and thus served as a protective factor against PTSD. However, previous studies provide some support to the neuroplastic compensation hypothesis. The frontal cortex is one of the later-maturing brain regions, exhibiting considerable plasticity and gray matter changes even during adulthood^51^. In a twin study, gray matter differences were not observed between the combat-unexposed twins of veterans with PTSD and combat-unexposed twins of veterans without PTSD; however, significant differences in the ACC were observed between veterans with and without PTSD, indicating that gray matter alteration in ACC could be the consequence of exposure to stress^52^. In a longitudinal study including survivors of a subway fire, greater prefrontal cortical thickness in trauma-exposed individuals was observed shortly after trauma, and the thickened prefrontal cortex gradually normalized over time until between-group differences disappeared approximately 4 years after trauma^19^. An association between prefrontal cortical thickness and the genotype of a gene related to cortical plasticity was also reported in that study^19^. Similar to the abovementioned studies, the increased thickness of the right mPFC observed in TECs in the current study might have resulted from traumatic experiences. If this causal relationship is assumed, mPFC thickening in traumatized refugees may persist for many years after the onset of trauma, due to repetitive traumatic events and ongoing stressors that refugees may continuously encounter.

In contrast to the findings in TECs, correlations were not found between psychological symptoms and mPFC thickness in our refugees with PTSD or HCs. This finding may be supported by the hypothesis that mPFC thickness increases as a compensatory mechanism after or during trauma, thereby helping with resistance against PTSD acquisition and a reduction in psychological symptoms. If a thicker mPFC is the result of trauma exposure, non-traumatized controls would not develop compensatory mPFC thickening, and the thickness would also not be associated with psychological symptoms. In addition, assuming that the compensatory mechanism hypothesis is correct, refugees with PTSD may have insufficient trauma-induced mPFC thickening to prevent the development of PTSD, and their mPFC thickness may not have an effect on psychological symptom severity.

The present study had several limitations. First, differences in age existed between groups. Although all analyses were adjusted for age to rule out the effects of age differences, the correction might have been insufficient; this could limit the interpretation of cortical thickness comparisons among groups. However, the linear regression slope for age *vs.* mPFC thickness derived from the current data was in the range of previously reported slopes for age-related cortical thinning in mPFC regions^53^. Because the mean mPFC thickness of each group was corrected for age using this slope, the correction for age was presumably adequate. Second, qualitative and detailed trauma characteristics (e.g., trauma severity, duration, number of traumatic experiences, and exact time since trauma) were not assessed in the current study. However, these specific measures are difficult to assess because the traumas encountered by refugees are often chronic and repetitive, thus creating difficulty in remembering each event separately. It could also be difficult to determine whether some events were sufficiently severe to be considered traumatic. In these instances, counting the number of trauma types experienced may be the reasonable alternative for estimating trauma burden. Third, TECs experienced fewer types of trauma than did participants in the PTSD group; this could explain why the TEC group did not acquire PTSD and manifested milder psychological symptoms. However, the difference in cortical thickness among groups, which is the main finding of the current study, would not have been affected by the difference in the number of trauma types encountered among groups, given the lack of correlation between the two variables. Future research is required to confirm whether a thicker mPFC helps to protect trauma-exposed individuals from the acquisition of PTSD and other psychological symptoms. Fourth, the recruitment of South Korean residents as HCs might have added nonspecific effects to the analysis. In particular, malnutrition is more prevalent in North Korean refugees than in South Korean residents, which might have impacted cortical thicknesses in the refugee groups. However, this potential effect might have reversed soon after refugee resettlement in South Korea, because refugees generally have adequate access to a nutritious diet after resettlement in South Korea. This assumption is supported by the previous finding that nutritional improvements rapidly reverse cortical thinning in anorexia nervosa patients^54,55^. Moreover, South Koreans share the same genetic background, history, and language with North Korean refugees. Therefore, nonspecific differences in cortical thickness between North Korean refugees and South Korean controls were presumed to be relatively small. Considering that recruiting North Korean residents is impossible and recruiting North Korean refugees without any traumatic experiences is very difficult, recruiting South Korean residents may be the best alternative. Fifth, medication use was not assessed in HCs, and the PTSD and TEC groups exhibited some differences in medication use status. However, HCs were presumably not on any psychotropic medication because psychiatric illnesses were ruled out in this group. Given that the TECs and HCs both did not use psychotropics, the significant cortical thickness difference between the two groups would not have been affected by this factor. Sixth, the current study was cross-sectional; therefore, causal relationships could not be determined. However, based on previous studies, we speculate that compensatory mechanisms in response to traumatic experiences might have caused differences in cortical thickness among the groups. Future longitudinal studies are required to determine causal relationships. Seventh, the recruitment of a specific group of refugees and the small proportion of male participants may limit the generalizability of the current findings to other traumatized individuals. However, our North Korean refugee participants reported experiencing traumatic events that are commonly experienced by refugees in general^15^. The timing of traumatic exposures in our sample was also similar to those described by other refugees, beginning from residency in their home country and extending throughout the flight process^15^. This overlap in trauma characteristics suggests that the findings of the current study could also have implications for other refugee groups. Nonetheless, generalization of the current findings to other traumatized populations requires substantial caution. Eighth, self-report measures were used in the current study, which could have led to bias and masked significant correlations with cortical thickness. Future studies using objective measurement of emotions (e.g., skin conductance, electroencephalography, or electromyography)^56^ may further clarify the findings of the current study. Finally, a liberal cluster-forming threshold (i.e., *P* < 0.05) was used in the group analysis of the current study. However, nonparametric permutation test was used to correct for multiple comparisons, which has shown adequate control for false positive rates even with lenient cluster-forming thresholds^57^.

In summary, North Korean refugees without PTSD had increased right mPFC thickness after traumatic experiences. The increased thickness of the right mPFC was associated with decreased anxiety, depression, and somatization. The increased mPFC thickness in traumatized refugees in the current study might have helped with preventing the development of PTSD and reducing psychological symptoms, and likely reflects neurobiological resilience achieved by potentially enhancing emotional regulation in the mPFC.

## Supplementary Information

Supplementary Information.

## Data Availability

The datasets analyzed during the current study are available from the corresponding author on reasonable request.
