# Supplementary material for: Increased medial prefrontal cortical thickness and resilience to traumatic experiences in North Korean refugees
Source: Sci Rep. 2021 Jul 21;11:14910. doi: 10.1038/s41598-021-94452-6 (PMC8295347; doi:10.1038/s41598-021-94452-6)
Supplement: Supplementary file 1 — Supplementary Information. [file 41598_2021_94452_MOESM1_ESM.docx]

**Title:** Increased medial prefrontal cortical thickness and resilience to traumatic experiences in North Korean refugees

**Authors:** Hyunwoo Jeong^1^, Yu Jin Lee^2^, Nambeom Kim^3^, Sehyun Jeon^4^, Jin Yong Jun^5^, So Young Yoo^6^, So Hee Lee^6^, Jooyoung Lee^7^, Seog Ju Kim^7^*

**Affiliations**

^1^Geumsan-gun Public Health Center, Geumsan, Republic of Korea

^2^Department of Psychiatry and Center for Sleep and Chronobiology, Seoul National University Hospital, Seoul, Republic of Korea

^3^Neuroscience Research Institute, Gachon University, Incheon, Republic of Korea

^4^Department of Psychiatry, Korea University College of Medicine, Korea University Anam Hospital, Seoul, Republic of Korea

^5^Department of Psychiatry, Seoul National Hospital, Seoul, Republic of Korea

^6^Department of Psychiatry, National Medical Center, Seoul, Republic of Korea

^7^Department of Psychiatry, Sungkyunkwan University School of Medicine, Samsung Medical Center, Seoul, Republic of Korea

**Supplementary Table S1.** Psychiatric comorbidities of the participants

| **Psychiatric comorbidities** | **PTSD group (n = 27)** | **Trauma-exposed controls  (n = 23)** | **Healthy controls  (n = 51)** | **p-value** |
| --- | --- | --- | --- | --- |
| Anxiety disorders (n) (%) | 6 (22.2%) | 1 (4.3%) | 0 (0%) | 0.001 |
| Depressive disorders (n) (%) | 11 (40.7%) | 1 (4.3%) | 0 (0%) | <0.001 |
| Somatic symptom and related disorders (n) (%) | 8 (29.6%) | 1 (4.3%) | 0 (0%) | <0.001 |
| Adjustment disorders (n) (%) | 3 (11.1%) | 1 (4.3%) | 0 (0%) | 0.04 |
| Feeding and eating disorders (n) (%) | 1 (3.7%) | 0 (0%) | 0 (0%) | 0.50 |

Abbreviations: PTSD: post-traumatic stress disorder
